# OTME-16. Polio virotherapy of murine brain tumors causes microglia/macrophage proliferation and inflammation that is potentiated by immune checkpoint blockade

**DOI:** 10.1093/noajnl/vdab070.067

**Published:** 2021-07-05

**Authors:** Yuanfan Yang, Michael Brown, Kevin Stevenson, Giselle López, William Kornahrens, Gao Zhang, Matthias Gromeier

**Affiliations:** Duke University Medical Center, Durham, NC, USA

## Abstract

PVSRIPO is a novel viral immunotherapy that has shown evidence of efficacy in a phase I clinical trial for recurrent GBM, resulting in 21% survival rate at 36 months following treatment. To improve clinical response rate, it is critical to resolve the mechanisms of action and therapy resistance *in vivo*, thereby designing effective combination therapy strategies.

We used immunocompetent mouse models of glioma (CT2A) and metastatic melanoma (B16) to dissect early and late events following virotherapy with PVSRIPO. A blinded systematic review of the pathology from 62 intracranial tumors, collected on different days following PVSRIPO (or control) treatment, was performed. An overall treatment effect, measured by tumor shrinkage, dis-cohesive growth pattern, microglia enrichment, was present in 88% of tumors on day 8, but the tissue response rate fell to 42% on days 10 & 12, and 14% on day 15. The control group showed no treatment effect throughout. RNAseq from the same set of samples showed acute induction of type-I interferon-related inflammation that faded with time in Gene Set Enrichment Analysis. This suggests that sustaining adaptive antitumor immunity elicited by immediate intratumor type-I IFN-dominant inflammation is critical to long term remission. Careful review of the post treatment pathology revealed an early enrichment of both T cells and microglia in the tumor microenvironment with a high Ki-67 proliferation index. We propose that the PVSRIPO therapy effect is dependent on macrophage/microglia mediated cellular immune response, likely in response to direct viral infection. This suggests potential therapeutic interventions, including blockade of the PD1:PD-L1 immune checkpoint, to potentiate antitumor CD8+T cells in response to PVSRIPO therapy. Indeed, combination therapy with αPD-L1 antibody in the CT2A model showed higher long term remission (37%, n=11), compared to either monotherapy; this effect is CD8+T cell- and macrophage-dependent, demonstrated by depletion studies *in vivo*.

